# Minilaparoscopic lumbar sympathectomy with 3 mm instruments for plantar hyperhidrosis

**DOI:** 10.1590/1677-5449.180072

**Published:** 2020-05-08

**Authors:** Marcelo Loureiro, Arlindo Nascimento de Lemos, Paolo Rogerio Oliveira Salvalaggio, Mohammad Alwazzan

**Affiliations:** 1 Universidade Positivo, Programa de Pós-graduação em Biotecnologia, Curitiba, PR, Brasil.; 2 Universidade Estadual de Campinas – UNICAMP, Departamento de Radiologia, Campinas, SP, Brasil.; 3 Faculdade São Leopoldo Mandic, Departamento de Cirurgia Vascular Campinas, SP, Brasil.; 4 Hospital Israelita Albert Einstein, São Paulo, SP, Brasil.; 5 Amiri Hospital, Department of Surgery, Kuwait.

**Keywords:** sympathectomy, endoscopic lumbar sympathectomy, plantar hyperhidrosis, simpatectomia, simpatectomia lombar endoscopica, hiperidrose plantar

## Abstract

Severe palmoplantar hyperhidrosis affects about 1.5-2.8% of the general population. Plantar hyperhidrosis (PHH) is related to foot odor, cold feet, skin lesions and infections, and even instability when walking. Endoscopic Lumbar Sympathectomy (ELS) is the treatment of choice for this condition. However, few surgeons have used this technique over the past 20 years because of its technical difficulty. Two and 3 mm instruments, rather than the standard 5 mm instruments, have been used to improve the results of several standard laparoscopic procedures. Use of these minilaparoscopic instruments to perform ELS so far has not yet been published. We describe a technique for ELS using minilaparocopic instruments, which we have used for our last 70 cases and has become our standard technique. The aim of this study is to demonstrate the feasibility of this technique and its advantages compared to the conventional technique.

## Resumo

A hiperidrose palmoplantar grave afeta cerca de 1,5-2,8% da população geral. A hiperidrose plantar está relacionada a odor dos pés, pés frios, lesões cutâneas, infecções, e até instabilidade da marcha. A simpatectomia lombar endoscópica (*endoscopic lumbar sympathectomy*, ELS) é o tratamento de escolha para essa condição; entretanto, tem sido utilizada por poucos cirurgiões nos últimos 20 anos, devido à sua dificuldade técnica. Instrumentos de 2 e 3 mm em vez de 5 mm vêm sendo utilizados para melhorar os resultados de vários procedimentos laparoscópicos padrão. O uso desses instrumentos para realizar ELS ainda não foi descrito. Descrevemos a técnica para ELS usando microinstrumentos, a qual vem sendo usada para os nossos últimos 70 casos, pois passou a ser nosso procedimento padrão. O objetivo deste estudo é apresentar a experiência com essa modificação técnica, assinalando as vantagens em relação à técnica tradicional.

## INTRODUCTION

Severe palmoplantar hyperhidrosis affects about 1.5%-2.8% of the general population.^1^^,^^2^ Palmar hyperhidrosis is obviously much more noticeable in affected individuals than plantar hyperhidrosis (PHH), but the latter can be just as socially and functionally disturbing as palmar hyperhidrosis, because it affects an area covered by shoes and other garments. Whether moderate or severe, cases of hyperhidrosis (both palmar and plantar) can pose functional and social problems.

Many options for treatment of PHH have been tried (oral oxybutynin, iontoforesis, topical agents, and botulin toxin injections), but without long term success for the majority of patients. None of these options compare to the effectiveness of surgical resection of the lumbar sympathetic chain to achieve complete control of excessive plantar sweating.^3^^,^^4^

From the outset of video laparoscopic surgery, in the late 1980s, many previously open abdominal operations were performed using the new technique. Retroperitoneal surgeries followed this trend. Lumbar sympathectomy is one of the open surgical techniques that can be performed via the laparoscopic approach. In 1995, Hourlay et al.^5^ described the first series of retroperitoneal video laparoscopic lumbar sympathectomies (SLVR). From 2002 to the present, many endoscopic (laparoscopic) approaches were developed for treatment of plantar hyperhidrosis in a number of European and also some South American countries.

Interest in Lumbar Sympathectomy performed via minimally invasive access has increased among patients who are affected by PHH as well as among physicians involved in the treatment of HH.

Surgeons are always looking for ways to improve results. Substitution of standard 5mm laparoscopic instruments with 3 mm instruments has already been proven to give better results for other laparoscopic operations in terms of surgical performance and aesthetic skin results.^6^

The aim of this paper is to describe the details of the technique and discuss our experience with these instruments.

## TECHNIQUE AND RESULTS

**Figure 1:**
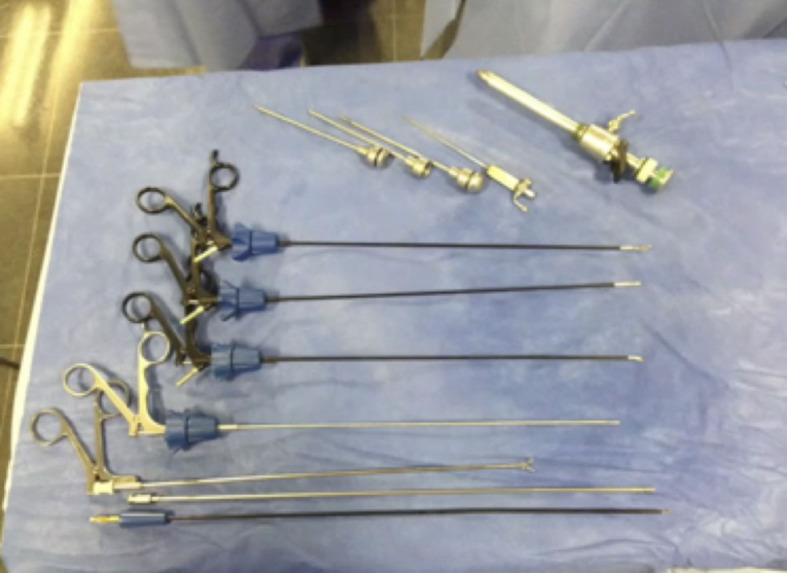
Needlescopic table set.

All procedures were carried out under general anesthesia and endotracheal intubation. The surgeon, the assistant surgeon, and the scrub nurse should be positioned on the same side of the operative target. The screen is on the opposite side, facing them. The patient is placed in a supine position with hyperextended flank and arms alongside the body. A table is prepared with the set of 3mm instruments (Figure 1).

Retroperitoneal space access is achieved with a combination of laparoscopy and retroperitoneal-guided trocar insertion.

The first step is laparoscopic access to the peritoneal cavity through the umbilicus. The laparoscope is directed to the flank where the retroperitoneal trocar will be placed. At that point, the initial skin incision is made and then a blunt dissection is performed until the pre peritoneal tissue is identified under laparoscopic vision.

Next, a 10mm trocar is placed and advanced up to that specific pre-peritoneal level, still under laparoscopic vision.

Once the retroperitoneal trocar is in place, the inflation tube is repositioned from the umbilical port to the retroperitoneal port. Once the 10 mm scope is in place, the retroperitoneal space is gently dissected first with the optics. Then two 3 mm ports are inserted under vision and the space is further developed. These ports allow introduction of the 3mm instruments (graspers, scissor, and hook).

The transversalis fascia is dissected, reaching the psoas muscle, which is the most important landmark in this space.

Care must be taken to keep in contact with the psoas and avoid the wrong plane of the Quadratus Lumborum.

**Figure 2:**
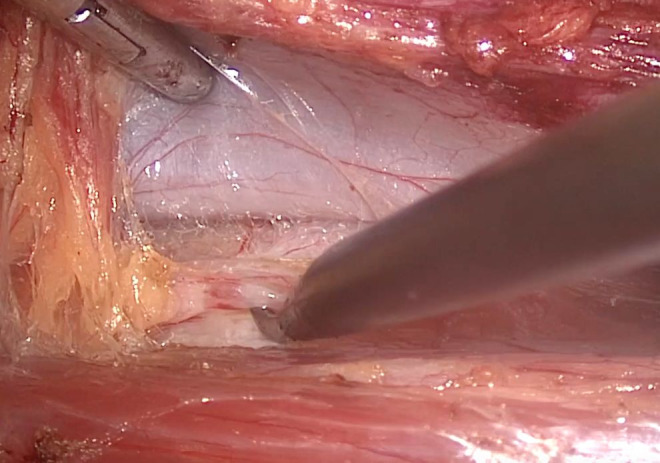
Lumbar chain dissection near to Inferior Cava Vein.

**Figure 3:**
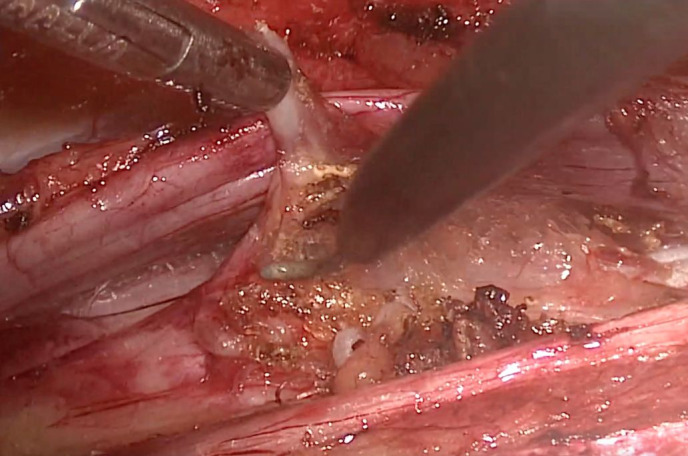
Resection of one lumbar ganglion.

Dissection continues as far as the lumbar vertebra. Just before it, on the left side, we can find the aorta and iliac arteries, and on the right side, the vena cava. The right sympathetic lumbar chain is completely covered by the vena cava and has to be dissected from it (Figure 2). Then it is resected within at least one lumbar ganglion (L2 or L3) (Figure 3).

We do not use the vertebra as anatomical landmark, but the inferior pole of the kidney and the navel. The rationale behind this is that a perpendicular approach, coming from the flank to the vertebral column will reach the targeted segment of the sympathetic nerve. The anhidrotic effect is achieved when one major ganglion is resected below the first ganglion.

Basically, the same technique is applied to both right and left sides. The right side is usually more complex because of the abundance of large lumbar veins, which can cause significant bleeding if damaged, and the need for vena cava dissection.

Operating time for simultaneous right and left lumbar sympathectomies is about 72 min (52 - 95). We used to take longer to perform this surgery before adopting micro instruments, but the improvement could possibly be better explained by the learning curve than by the choice of instruments.

Perioperative complications are rare, and those that occur are related to one of the following situations: opening of the peritoneal tendon that supports the extra-peritoneal space (5 out of 70); misidentification of the sympathetic trunk (0); inadvertent lesion of lymphatic ducts (5), and bleeding from lumbar vessels (1). Although the right side is technically more difficult because of the vena cava, these complications were independent of the side of surgery. In comparison with the 97 patients we operated before routine use of mini-laparoscopy, we observed that opening of the peritoneum was much more common during our 5 mm trocar and grasper period (23 out of 97). We also had one genital femoral nerve resection because of misidentification of the lumbar chain, 3 small lumbar vessel lesions, and 15 lymphatic duct lesions (Table 1). Lymphatic duct dissection with postoperative accumulation of lymphatic fluid is another possible problem. It should be noted that small injuries to the lymphatic channels are usually of no consequence and are not a reason for aborting the procedure. Once the procedure is completed, and the retroperitoneal space is deflated, a small lymphatic leak resolves on its own.

**Table 1:** Perioperative complications between series.

**Perioperative complications**	**5 mm series (n=97)**	**3 mm series (n=70)**
Peritoneum opening	23	5
Misidentification	1	0
Lymphatic duct lesion	3	5
Lumbar vein lesion	15	1
Total	42	11

*n =* number of patients in each group.

Regarding recovery, it is usually uneventful and patients were generally discharged on the same day, or the day after. Only 3 patients were discharged on postoperative day 2, and none of the patients in the minilaparoscopic instruments series had to be reoperated.

Immediate control of PHH was achieved in all patients (140 feet, 100%).

Regarding postoperative complications, pain is in general very well tolerated, even though some patients (5-10%) experienced post sympathectomy neuralgia, a bothersome self-limited pain in the back and lower legs. Only 2 of them experienced pain for a longer period, exceeding 1 week. In some patients, uncomfortable pain may recur even after some weeks. We only use oral analgesics for postoperative pain management and reassure patients of its self-limited character. This has not been improved by adoption of the thinner instruments.

No retrograde ejaculation was observed among our male patients (5/70) and there were no sexual complaints either, as have been previously reported in other published experiences.

Follow-ups at 1week and 1 month after surgery revealed very convincing cosmetic results. This is probably the best advantage of using micro instruments together with better technical performance. Both are difficult to measure but seem to generally improve the results of ELS.

After an average follow-up of 1 year (3 to 38 months), no patients in this cohort had recurrence of excessive plantar sweating. Compensatory sweating was reported by just 7 patients (10%), in particular from the trunk, and only one of them considered it to be bothersome. All of these patients had previously undergone endoscopic thoracic sympathectomy (ETS) and in all of them compensatory sweating was already a complaint and did not increase significantly after ELS.

## DISCUSSION

Interest in lumbar sympathectomy performed via minimally invasive access has been increasing among patients who are affected by PHH, as well as among physicians involved in treating this disease. There are some slight technical differences described related to access to the retroperitoneal space, or use of clips instead of resection of the lumbar chain.^3^^,^^7^^,^^8^ Surgery with mini-instruments seems to increase the safety of ELS, at least by providing better visualization of the operative field. As previously reported for other minimally invasive surgical techniques, use of 3mm instruments adds some advantages. They have been used in other types of operations, such as bariatric, gallbladder, and hernia surgery.^6^^,^^9^^,^^10^

There are a number of different methods for performing ELS, since some surgeons have developed different approaches and techniques based on their preferences and experience. In view of the long learning curve, the authors recommend that a more experienced surgeon should accompany a beginner in this technique.

Bleeding from retroperitoneal vessels is very unusual during ELS. If it happens, the surgeon must try to control it with the instruments that are in place. If any difficulty worsens, a 5 or 10 mm trocar allows introduction of a larger and better aspiration device, and so a clip applier or suture device could be inserted. Conversion to open surgery is exceptional and did not occur in our series.

Peritoneal tears can make endoscopic retroperitoneal surgery very bothersome and difficult. Use of these thinner instruments seems to protect against this technical complication, because they are easier to move and identify inside the narrow, distended, retroperitoneal space. If tears occur, they can be controlled by inserting a Veress needle or a 5mm trocar into the intra-abdominal space to relieve pressure and allow work to continue in the retroperitoneal space. Depending on the size of the tear, one should go for a trans-peritoneal approach or even abort the procedure and reschedule it for one month later.

Minilaparoscopic techniques can add some interesting advantages to this procedure. This is considered a technically demanding operation. The sympathetic lumbar chain is in direct contact with important structures especially on the right side, where it must be separated from the inferior vena cava by delicate and meticulous movements. The micro instruments are very precise and allow better movements and better visualization of the operating field. There is also the benefit of smaller incisions with very subtle wound and scar formation, which is important from the patient’s point of view.

All of the authors state that there are no conflicts of interest regarding any information contained in this article.

## CONCLUSION

Minilaparoscopic lumbar sympathectomy is a safe procedure. Use of minilaparoscopic instruments could result in improved outcomes compared to standard laparoscopy.
